# Genetic Analysis of Patients With Early-Onset Parkinson’s Disease in Eastern China

**DOI:** 10.3389/fnagi.2022.849462

**Published:** 2022-05-11

**Authors:** Ping Hua, Yuwen Zhao, Qian Zeng, Lanting Li, Jingru Ren, Jifeng Guo, Beisha Tang, Weiguo Liu

**Affiliations:** ^1^Department of Neurology, Affiliated Nanjing Brain Hospital, Nanjing Medical University, Nanjing, China; ^2^Department of Neurology, Xiangya Hospital, Central South University, Changsha, China; ^3^National Clinical Research Center for Geriatric Disorders, Xiangya Hospital, Central South University, Changsha, China; ^4^Department of Geriatrics, Xiangya Hospital, Central South University, Changsha, China; ^5^Hunan Key Laboratory of Medical Genetics, Centre for Medical Genetics, Xiangya Hospital, School of Life Sciences, Central South University, Changsha, China; ^6^Key Laboratory of Hunan Province in Neurodegenerative Disorders, Central South University, Changsha, China

**Keywords:** Parkinson’s disease, early onset, whole exome sequencing, genetics, Chinese population

## Abstract

**Background:**

Genetic factors play an important role in the pathogenesis of early-onset Parkinson’s disease (EOPD). To date, more than 20 pathogenic genes associated with Parkinson’s disease (PD) have been identified. This study aims to explore the mutation spectrum of EOPD and the clinical characteristics of mutation carriers in eastern China.

**Methods:**

We recruited 155 unrelated EOPD patients, including 8 familial and 147 sporadic EOPD (age at onset ≤ 50 years). Overall, 24 known PD-associated genes were detected by whole exome sequencing and multiplex ligation-dependent probe amplification (MLPA) from patient samples. The genetic and clinical characteristics of pathogenic/likely pathogenic (P/LP) loci in this cohort were analyzed.

**Results:**

Overall, 14 (9.03%) patients were detected with P/LP variants distributed in seven genes. The most frequent mutation occurred in *PRKN* (7/155, 4.52%), followed by *LRRK2* (2/155, 1.29%), *SNCA*, *CHCHD2*, *TMEM230*, *DNAJC13* and *PLA2G6* (1/155, 0.64%, respectively). Exon rearrangement mutations accounted for 57.9% (11/19) of all mutations in *PRKN*. Four novel variants were detected: c.14T > C (p.M5T) in *SNCA*, c.297C > A (p.Y99X) in *CHCHD2*, c.2578C > T (p.R860C) in *DNAJC13* and c.4C > T (p.Q2X) in *TMEM230*. We found the first case of *LRRK2* c.6055G > A (p.G2019S) mutation in Chinese population. The median onset age of patients with P/LP mutations in autosomal recessive genes (*PRKN* and *PLA2G6*) was about 18.0 years earlier than patients without mutation. The proportion of patients with mutations were 63.64%, 27.03% and 9.68% when patients were stratified according to the age of onset at ≤ 30, ≤ 40 and ≤ 50 years, respectively.

**Conclusion:**

Early-onset Parkinson’s disease patients from eastern China present a regional specific mutation spectrum. Analysis of larger patient cohorts is required to support these findings, and mechanistic studies of the four novel missense/non-sense mutations will clarify their role in the pathogenicity of EOPD.

## Introduction

Parkinson’s disease (PD) is a progressive neurodegenerative disease affecting 1% of people older than 60 years and 4% of people older than 85 years (Kalia and Lang, 2015). With aging of the population, prevalence is expected to exceed 5 million in China by 2030, accounting for almost half of the global number of patients with PD (Li et al., 2019). Parkinson’s disease involves complex pathogenic mechanisms, with advancing age as the greatest risk factor; both environmental and genetic factors also affect disease risk and progression. Approximately 5%–10% of patients develop PD early and display different clinical characteristics from PD in the elderly (Mehanna et al., 2014). Thus, PD is divided into early-onset Parkinson’s disease (EOPD) and late-onset Parkinson’s disease (LOPD) according to the age of onset. There is no unified international standard for defining the age of onset for EOPD. However, age boundaries have a significant impact on the statistical association of genetic mutations to PD. Taking the *PRKN* gene as an example, several European studies defining EOPD onset age at 45 found that more than 15% of sporadic EOPD patients carried pathogenic mutations in *PRKN* (Lücking et al., 2000; Periquet et al., 2003), while a Chinese study defining EOPD onset at 50 found only 2.66% of sporadic EOPD patients with such mutations (Zhao et al., 2020). At present, most studies set the age of onset for EOPD at ≤50 years old based on the genetic evidence from previous studies (Tanner et al., 1999). Genetic factors are closely related to EOPD. The earlier the onset age, the greater the possibility of detecting genetic variation (Kasten et al., 2018; Blauwendraat et al., 2020a,b; Zhao et al., 2020). Screening the pathogenic gene mutation spectrum in EOPD patients will help us to understand the pathogenesis of PD and explore targeted treatment strategies.

A growing number of pathogenic genes for PD have been identified and assigned *PARK* numbers according to their reported order (Lunati et al., 2018). Parkinson’s disease caused by pathogenic variants of these genes comply with Mendelian genetics, and are referred to as monogenic Parkinson’s disease. The rare pathogenic variants of these genes result in 20% of EOPD and no more than 3% of LOPD (Klein and Westenberger, 2012). The *PRKN*, *PINK1* and *DJ-1* genes are the three most common genes of autosomal-recessive EOPD, and account for approximately 13% of EOPD occurring before the age of 40 years (Puschmann, 2013). The *SNCA*, *LRRK2* and *VPS35* are autosomal-dominant genes with relatively clear pathogenic mechanisms (Lunati et al., 2018; Kluss et al., 2019; Cutillo et al., 2020). The significance of other genes requires further functional validation and replication in larger cohorts and different populations. These genes include *PLOG*, *GBA*, *LRP10*, *RIC3*, and *RAB39b*; suggesting a wide mutational spectrum for EOPD. Numerous studies around the world from Germany, Britain, Finland and South Korea, as well as southwest China, western China and Taiwan have revealed the genetic heterogeneity of different regions and races for EOPD (Siitonen et al., 2017; Lin et al., 2019; Tan et al., 2019; Trinh et al., 2019; Youn et al., 2019; Li et al., 2020; Zhao et al., 2020). At present, there is no genetic map of EOPD patients in eastern China.

To provide insights into the mutational profiles of eastern Chinese EOPD patients, this study screened mutations in 24 known PD-related genes through whole exome sequencing (WES) and multiplex ligation-dependent probe amplification (MLPA) in this cohort. In addition, the clinical characteristics of patients with pathogenic/likely pathogenic (P/LP) variants were analyzed to provide a correlation between genotype and phenotype.

## Materials and Methods

### Subjects

A total of 155 patients with EOPD (age at onset ≤ 50 years) including 147 sporadic PD; 5 from families with autosomal-dominant (AD) PD and 3 from families with autosomal-recessive (AR) PD; were recruited from the Center for Parkinson and Movement Disorders at Nanjing Brain Hospital between September 2009 and June 2018. Among these patients, 136 cases came from the Jiangsu Province, while 19 cases came from the southern region of Anhui Province. Data from these patients were added into Parkinson’s Disease & Movement Disorders Multicenter Database and Collaborative Network in China (PD-MDCNC), established by Tang’s team. All the patients were diagnosed by two experienced neurologists, based on the United Kingdom PD Society Brain Bank diagnostic criteria for PD (Gibb and Lees, 1988) or the 2015 Movement Disorder Society clinical diagnostic criteria for PD (Postuma et al., 2015). Exclusion criteria included other neurodegenerative disorders, psychiatric disorders, and severe physical illnesses. Motor symptoms were evaluated by the Unified Parkinson’s disease Rating Scale (UPDRS) Parts I and II, and Hoehn-Yahr staging (H-Y). Non-motor symptoms were assessed by the 24-item Hamilton Rating Scale for Depression (HAMD), 14-item Hamilton Rating Scale for Anxiety (HAMA), Parkinson’s Disease Sleep Scale (PDSS), Mini-Mental State Examination (MMSE), Montreal Cognitive Assessment (MoCA), and Non-Motor Symptoms Screening Questionnaire (NMSQ). Medication was recorded and converted to the levodopa equivalent daily dose (LED) (Tomlinson et al., 2010).

### Genetic Analysis

#### Whole-Exome Sequencing

Genomic DNA was isolated from citric acid anticoagulated peripheral blood leukocytes using the DNA Extraction Kit (Tiangen, Beijing, China). The exon and flanking regions of 24 PD-associated genes (Supplementary Table 1) were captured as previously described (Zhao et al., 2020). Whole-exome sequencing (WES) was performed on the Illumina HiSeq X10 platform with 150bp paired-end reads. The acquired reads were mapped onto the reference human genome (UCSC hg19) using the Burrow-Wheeler Aligner. Quality control and annotation were done as previously described (Zhao et al., 2020). Rare variants were filtered according to the minor allele frequency (1% for recessive genes; 0.1% for dominant genes) in East Asian population from public databases (GnomAD and ExAC).

#### Multiplex Ligation-Dependent Probe

Large exon deletions or duplications (copy-number variants, CNVs) of common PD-associated genes including *SNCA*, *PRKN*, *PINK1*, *DJ-1*, *ATP13A2*, and *LRRK2* were detected using the Salsa MLPA kit P051-C1 (MRC-Holland, Amsterdam, The Netherlands).

#### Bioinformatics Analysis

The pathogenicity of missense mutations, non-sense variants, splicing site variants and Indels screened by WES was predicted by CADD,^1^ PolyPhen-2,^2^ SIFT,^3^ Mutation Taster^4^ and ReVe (Li et al., 2018). We also searched pathogenic variants in Pubmed,^5^ Gene4PD,^6^ and MDSGene,^7^ to determine whether they were reported before. According to the American College of Medical Genetics and Genomics (ACMG) recommendations (Richards et al., 2015), the candidate variants were further interpreted and classified into pathogenicity (P), likely pathogenicity (LP), uncertain significance (US), likely benign (LB) and benign (B). For pathogenic or likely pathogenic (P/LP) variants, patients carrying two or more allele variants and their first-degree relatives were sequenced by Sanger and/or MLPA for co-separation analysis.

### Statistical Analysis

Statistical analysis was performed using the SPSS software, version 18.0. Quantitative data with, or near, normal distribution were described by mean ± standard deviation, and *t*-test was used for comparison between the groups. Quantitative data with skewed distribution were described by median (minimum-maximum), and Mann-Whitney non-parametric test was used for comparison between the groups. The demographic and clinical data were compared between the P/LP variant carriers and non-carriers, and between carriers with AR/AD genetic variants and non-carriers. The frequency of gene mutations at different age-of-onset was compared. A two-tailed P < 0.05 was considered statistically significant.

## Results

### Demographic and Clinical Information

The mean age at onset of the 155 patients was 43.39 ± 7.13 years, 58.7% were male; the mean age at assessment was 50.76 ± 9.28 years; and the mean duration of disease at assessment was 8.16 ± 7.40 years. The medium H-Y score was 2.0, ranging from 1.0 to 5.0, and 65% of patients were in the early stages of the disease. The motor and non-motor symptoms were relatively mild (Table 1).

**TABLE 1:** Clinical data of 155 patients with EOPD.

Clinical data	EOPD
	(*n* = 155)
Male [case (%)]	91(58.7%)
Age at onset (year)	43.39 ± 7.13
Age at assessment (year)	50.76 ± 9.28
Duration at assessment (year)	6.0 (1–50)
LED (mg/d)	533.7 ± 289.3
UPDRS-I score	3.59 ± 2.52
ADL score	12.67 ± 7.39
UPDRS-III score	28.69 ± 16.83
H-Y staging	2.0 (1.0–5.0)
MMSE score	27.49 ± 2.44
MoCA score	23.71 ± 4.95
HAMD score	11.18 ± 7.25
HAMA score	7.48 ± 5.18
PDSS score	120.07 ± 23.47
NMSQ score	9.25 ± 5.16

*EOPD: early-onset Parkinson’s disease; LED: levodopa equivalent daily dose; UPDRS: Unified Parkinson’s disease rating scale; ADL: Activities of Daily Living; H-Y: Hoehn-Yahr staging; MMSE: Mini-Mental State Examination; MoCA: Montreal Cognitive Assessment; HAMD: 24-item Hamilton Rating Scale for Depression; HAMA: 14-item Hamilton Rating Scale for Anxiety; PDSS: Parkinson’s Disease Sleep Scale; NMSQ: Non-Motor Symptoms Screening Questionnaire.*

### Genetic Analyses

#### P/LP Variants

P/LP mutations were detected in 14 (9.03%) patients, including one patient with family history of ADPD (1/5, 20%), one patient with family history of ARPD (1/3, 33.3%), and 12 sporadic patients (12/147, 8.8%) (Table 2). Among these 14 patients, immediate relatives of seven patients came for verification, and the genotypes of two patients’ parents could be inferred from their genotypes, so the pedigree chart of nine families is shown in Supplementary Figure 1.

**TABLE 2:** List of 14 patients carrying rare P/LP variants in PD associated genes.

No.	Sample ID	Gender	OA	Gene	Nucleotide change	A.A. alteration	Exonic Func.	Hom/Het	Mode of Inheritance	ACMG	Reported
1	4309	M	23	*PRKN*	c.850G > C	p.G284R	non-syn	Hom	AR	LP	Y
2	4328	M	19	*PRKN*	c.850G > C	p.G284R	non-syn	Hom	AR	LP	Y
				*PRKN*	Exon5-7 del		CNV	Het	AR	P	Y
3	4349	M	26	*PRKN*	Exon2-3 del		CNV	Het	AR	P	Y
				*PRKN*	Exon5 del		CNV	Het	AR	P	Y
4	4351	M	48	*PRKN*	Exon2-3 dup		CNV	Het	AR	P	Y
				*PRKN*	Exon4 dup		CNV	Het	AR	P	Y
5	4354	F	28	*PRKN*	c.1079G > T	p.C360F	non-syn	Het	AR	LP	Y
				*PRKN*	Exon3-5 del		CNV	Het	AR	P	Y
6	4364	M	28	*PRKN*	c.850G > C	p.G284R	non-syn	Het	AR	LP	Y
				*PRKN*	Exon3-4 del		CNV	Het	AR	P	Y
7	4427	M	37	*PRKN*	Exon6 del		CNV	Het	AR	P	Y
				*PRKN*	Exon3-4 del		CNV	Het	AR	P	Y
8	4355	M	33	*PLA2G6*	c.1634A > G	p.K545R	non-syn	Het	AR	LP	Y
				*PLA2G6*	c.991G > T	p.D331Y	non-syn	Het	AR	LP	Y
9	4390	M	48	*LRRK2*	c.6055G > A	p.G2019S	non-syn	Het	AD	LP	Y
10	4339	M	30	*LRRK2*	c.4339G > A	p.V1447M	non-syn	Het	AD	LP	Y
11	4292	F	50	*CHCHD2*	c.297C > A	p.Y99X	stopgain	Het	AD	P	N
12	4356	M	35	*DNAJC13*	c.2578C > T	p.R860C	non-syn	Het	AD	LP	N
13	4375	F	50	*SNCA*	c.14T > C	p.M5T	non-syn	Het	AD	LP	N
14	4392	M	50	*TMEM230*	c.4C > T	p.Q2X	stopgain	Het	AD	P	N

*OA: onset age; ACMG: American College of Medical Genetics and Genomics; P: pathogenic; LP: likely pathogenic; non-syn: non-synonymous mutation; CNV: Copy number variants; Hom: Homozygous; Het: Heterozygous.*

*Patient 3 and patient 10 have an ARPD family history; other 12 patients were sporadic.*

*PRKN* was the most prevalent causative gene in the 155 patients with EOPD (7/155, 4.52%). Among the seven P/LP-variants carriers, two had a homozygous variant (c.850G > C; one case also had a heterozygous deletion of exon 5–7), and five had compound heterozygous variants (Table 2). A compound heterozygous variant of *PLA2G6* (c.1634A > G/c.991G > T) was also identified. All variants for both genes had been reported previously (Koh et al., 2019b), and included in the MDSGene and Gene4PD databases. Two LP variants of the *LRRK2* were detected in this group, accounting for 1.3% of the total patients. One case was c.4339G > A (p.V1447M), which has been reported previously (Zhao et al., 2020). The other case detected was c.6055G > A (p.G2019S). This is the first case of *LRRK2* c.6055G > A (p.G2019S) mutation detected in the Chinese population. In addition, four heterozygous missense/non-sense mutations were found in the ADPD-related genes *CHCHD2*, *DNAJC13*, *SNCA* and *TMEM230*, which have not been reported previously (Table 3).

**TABLE 3:** Population frequency and pathogenicity prediction of four novel variants.

	*CHCHD2*	*DNAJC13*	*SNCA*	*TMEM230*
Nucleotide change	c.297C > A	c.2578C > T	c.14T > C	c.4C > T
A.A. alteration	p.Y99X	p.R860C	p.M5T	p.Q2X
Exonic Func.	Stopgain	non-syn	non-syn	stopgain
Hom/Het	Het	Het	Het	Het
Mode of Inheritance	AD	AD	AD	AD
gnomAD_exome_EAS	−	−	−	9.96E-05
ExAC_EAS	−	−	−	−
CADD	43:D	34:D	15.00:T	23.6:D
ReVe	0.763:D	0.997:D	0.806:D	0.265:T
SIFT	−:−	0.0:D	0.103:T	−:−
PolyPhen2-HVAR	−:−	0.994:D	0.922:D	−:−
Mutation taster	1:D (NMD)	1:D	0.999:D	1:D (NMD)
PhyloP	5.919:Conserved	5.795:Conserved	2.147:Conserved	0.163:Non-conserved
PhastCons	1:Conserved	1:Conserved	1:Conserved	0.002:Non-conserved

*Non-syn: non-synonymous mutation; Het: Heterozygous; D: deleterious; T: tolerable; NMD: non-sense mediated mRNA decay.*

#### Non-pathogenic Variants

There were 22 patients that had non-pathogenic variants, including four variants of AD genes (Supplementary Table 2) that were classified as uncertain significance according to the ACMG recommendations (the population frequency and pathogenicity prediction of these four variants see in Supplementary Table 3), and 19 single heterozygous variants of AR genes (one patient carried two simple heterozygous variants; see details in Supplementary Table 4). Although a single heterozygous variant of the AR gene is not genetically pathogenic, six variants (c.1285 + 2T > C, c.850G > C, deletion of exon 2–3, deletion of exon 8-9 of *PRKN*, p.R3296Gfs*2 frameshift mutation of *VPS13c*, and p.N801Rfs*11 frameshift mutation of *SYNJ1*) among these 19 single heterozygous variants need to be paid attention to as these six variants may cause coding abnormalities of single chromosomes and become pathogenic, if they exist in homozygous form. Among them, the patient with c.1285 + 2T > C of *PRKN* has a history of ARPD. We failed to get the families of these patients to do co-separation analysis, otherwise it may help us explore whether these heterozygous variants increase the risk of PD.

### Clinical Phenotypes of P/LP Variants

Clinical phenotypes were compared between carriers of P/LP variants and non-carriers, and between carriers of US variants and non-carriers (Table 4). The results showed that the median age at onset of patients with P/LP variants (mean 36.07 years; median 34 years) was 12 years earlier than that of patients without these gene variants (mean 44.52 years; median 46 years). However, there was no significant difference in the severity of motor and non-motor symptoms between these two groups. Comparing the US variant carriers and non-carriers, there was no significant difference in age of onset of clinical manifestation.

**TABLE 4:** Comparison of clinical phenotypes between P/LP variant carriers and non-carriers, and between with non- pathogenic variant carriers and non-carriers.

Clinical features	Non-carriers	P/LP variant carriers	*P*	Non-pathogenic variant carriers	*P*
	(*n* = 122)	(*n* = 14)		(*n* = 19)	
Age at onset (year)*	44.48 ± 5.76	36.07 ± 11.13	0.033	41.74 ± 8.35	0.144
Male (case,%)	71(58.2%)	11(78.6%)	0.271	9 (47.4%)	0.372
Age at assessment (year)*	51.61 ± 8.18	44.06 ± 13.29	0.158	50.19 ± 10.85	0.825
Disease duration (year)*	5.0 (1–50)	5.0 (2–31)	0.922	8.0 (2–23)	0.106
LED (mg/d)	515.0 ± 279.8	403.7 ± 268.9	0.409	720.1 ± 304.9	0.013
UPDRS-I score	3.48 ± 2.59	4.11 ± 2.21	0.401	4.07 ± 2.34	0.321
ADL score*	12.92 ± 7.22	12.89 ± 9.74	0.724	10.69 ± 7.39	0.219
UPDRS-III score	29.30 ± 16.17	28.00 ± 25.39	0.258	25.91 ± 13.66	0.423
H-Y*	2.10 ± 0.87	2.04 ± 1.12	0.539	2.09 ± 0.85	0.737
MMSE score*	27.54 ± 2.48	27.30 ± 2.71	0.817	27.53 ± 2.21	0.828
MoCA score*	23.49 ± 5.07	24.20 ± 5.03	0.5801	24.93 ± 4.39	0.267
HAMD score	11.08 ± 7.31	9.30 ± 5.81	0.603	12.00 ± 7.13	0.479
HAMA score*	7.54 ± 5.38	5.60 ± 3.31	0.347	7.50 ± 3.93	0.644
PDSS score	120.18 ± 21.99	131.67 ± 12.86	0.196	113.09 ± 34.79	0.590
NMSQ score	9.37 ± 5.41	8.29 ± 3.68	0.748	8.92 ± 3.90	0.865

*Mann-Whitney non-parametric test was used for non-normal distribution data.

*P/LP: pathogenic or likely pathogenic; LED: levodopa equivalent daily dose; UPDRS: Unified Parkinson’s disease Rating Scale; ADL: Activities of Daily Living; H-Y: Hoehn-Yahr staging; MMSE: Mini-Mental State Examination; MoCA: Montreal Cognitive Assessment; HAMD: 24-item Hamilton Rating Scale for Depression; HAMA: 14-item Hamilton Rating Scale for Anxiety; PDSS: Parkinson’s Disease Sleep Scale; NMSQ: Non-Motor Symptoms Screening Questionnaire.*

The age at onset and NMSQ score of patients with *PRKN* variants were significantly lower than those without mutation in this gene, and the course of disease at the time of evaluation was significantly longer than those without gene mutation. However, there was no significant difference in the severity of motor and non-motor symptoms between the two groups, indicating that disease progression in *PRKN* variant patients was relatively slower in the tested Chinese population (Table 5).

**TABLE 5:** Comparation of clinical phenotypes between carriers with P/LP variants in *PRKN* and non-carriers.

Clinical features	Non-carriers	*PRKN* P/LP variant carriers	*P*
	(*n* = 122)	(*n* = 7)	
Age at onset (year)*	44.48 ± 5.76	29.86 ± 9.72	0.001
Male (case,%)	71 (58.2%)	6 (75.0%)	0.360
Age at assessment (year)*	51.61 ± 8.18	41.64 ± 14.92	0.176
Disease duration (year)*	5.0 (1–50)	18. 5 (2–31)	0.025
LED (mg/d)	515.0 ± 279.8	349.0 ± 144.6	0.593
UPDRS-I score	3.48 ± 2.59	3.60 ± 3.29	0.678
ADL score*	12.92 ± 7.22	14.75 ± 9.08	0.821
UPDRS-III score	29.30 ± 16.17	35.20 ± 32.20	0.686
H-Y*	2.10 ± 0.87	2.40 ± 1.67	0.962
MMSE score*	27.54 ± 2.48	27.40 ± 3.29	0.859
MoCA score*	23.49 ± 5.07	24.40 ± 5.98	0.557
HAMD score	11.08 ± 7.31	9.00 ± 6.78	0.498
HAMA score*	7.54 ± 5.38	5.20 ± 4.32	0.372
PDSS score	120.18 ± 21.99	134.00 ± 16.97	0.383
NMSQ score	9.37 ± 5.41	6.00 ± 1.00	0.002

*Mann-Whitney non-parametric test was used for non-normal distribution data.

*P/LP: pathogenic or likely pathogenic; LED: levodopa equivalent daily dose; UPDRS: Unified Parkinson’s disease Rating Scale; ADL: Activities of Daily Living; H-Y: Hoehn-Yahr staging; MMSE: Mini-Mental State Examination; MoCA: Montreal Cognitive Assessment; HAMD: 24-item Hamilton Rating Scale for Depression; HAMA: 14-item Hamilton Rating Scale for Anxiety; PDSS: Parkinson’s Disease Sleep Scale; NMSQ: Non-Motor Symptoms Screening Questionnaire.*

In this cohort, the onset age of carriers with AD gene variants (mean 43.83 years, median 49 years) was older than carriers with AR gene variants (mean 30.25 years, median 28 years) (Supplementary Table 5). Three patients with variants in *CHCHD2*, *SNCA* and *TMEM230* were 50 years old, and two patients with variants in *LRRK2* were 30 and 48 years old, respectively. The median onset age of patients with the *PRKN* mutation was only 27 years, while the onset age of patients with *PLA2G6* mutation was 33 years. The molecular diagnosis rate of 155 patients with EOPD in this cohort was inversely proportional to the age at onset. In this sample, the rate of P/LP variants occurring in patients with onset age below 30 years was as high as 54.55% (6/11), reducing to 24.32% (9/37) in patients with onset age below 40, and the rate in patients with onset age below 50 was only 9.03% (14/155).

## Discussion

This study investigated the genetic causes of EOPD patients in eastern China using WES integrated with MLPA. The results identified 14 patients (9.03%) with P/LP variants in *PRKN*, *PLA2G6*, *LRRK2*, *SNCA*, *CHCHD2*, *TMEM230* and *GIGYF2*; the first case of *LRRK2* c.6055G > A (p.G2019S) mutation in a Chinese population, and four novel P/LP mutation sites. This revealed some geographical heterogeneities.

The proportion of familial PD in this study (5.2%) was lower than in the study conducted in southwest (19.6%) of China (Li et al., 2020). However, the proportion of patients carrying P/LP variants in this study was a bit higher than the study in southwest (7.5%). Additionally, the proportion of patients carrying the P/LP variants in the sporadic EOPD cohort in southcentral of China was only 4.59% (Zhao et al., 2020). The most common mutated gene across all three studies was *PRKN*, as it accounted for nearly 50.0% of all patients with identifiable mutations. The most common form of pathogenic mutation in *PR*KN was exon rearrangement with compound heterozygous mutation. We also found that the mean age of onset for patients with *PRKN* mutations was 15 years earlier than that of EOPD patients without mutations, and this is consistent with previous reports (Kasten et al., 2018; Zhao et al., 2020). In addition to *PRKN*, an LP variant in *PLA2G6* was also detected. The age of onset for this patient was 35 years, which was consistent with a previous report that patients with ARPD-related gene mutations had an early age of onset (Kasten et al., 2018). Previous studies have reported that patients with *PRKN*, *PLA2G6*, *PINK1* and *ATP13A2* mutations showed significantly earlier onset than patients with *VPS13C* and *RIC3* variants, while those with *LRRK2*, *SNCA* and *GIGYF2* mutations generally showed later onset (Zhao et al., 2020). Therefore, genetic testing can be helpful in diagnosing EOPD when symptoms occur before 40 years of age, especially for those with a family history of ARPD. In this regard, the MLPA technology is particularly useful in detecting exon rearrangement variations in EOPD patients, and should be considered for routine use in clinics.

It has been reported that the mutation frequency of *PINK1* is secondary to *PRKN*, and occurs in 3.7% of AR-EOPD patients (Hernandez et al., 2016). However, pathogenic variants of *PINK1* were not detected in our cohort, and one patient carrying the *PINK1* variant was detected in the study of southwest and southcentral regions, respectively. A compound heterozygous variant of *PLA2G6* (c.1634A > G, c.991G > T) was detected, which is the only mutation in a recessive gene detected in our study, aside from *PRKN*. The 33-year-old patient suffered from dystonia of the left foot, and developed dyskinesia after two years despite responding well to levodopa in early treatment, which was consistent with the clinical manifestation of dystonia-Parkinson’s syndrome caused by *PLA2G6* (Paisan-Ruiz et al., 2009). The study in southwest China also detected one compound heterozygous variant of *PLA2G6* (Li et al., 2020). The southcentral study detected three compound heterozygous variants of *PLA2G6* in sporadic EOPD, and was found to be the second most frequent mutation in the ARPD cohort (Zhao et al., 2020). It seems that the mutation frequency of *PLA2G6* in the Chinese population is higher than *PINK1* and *DJ-1*, and most of them exist in the compound heterozygous state. Moreover, several single heterozygous mutations of *PLA2G6* detected in Chinese PD patients were associated with impaired *PLA2G6* phospholipase activity (Gui et al., 2013). Of particular interest is mutations in *PLA2G6* have been linked to several neurological diseases including infantile neuroaxonal dystrophy (INAD) (Khateeb et al., 2006), neurodegeneration with brain iron accumulation (NBIA) (Morgan et al., 2006), hereditary spastic paraplegia (HSP) (Koh et al., 2019a) and PD, but the underlying mechanism is still unknown. Thus, further research on the complexity of gene-gene and gene-environment interactions will be required to unveil the role of *PLA2G6* in the Chinese population.

Four novel mutations were detected in this cohort, all of which were SNVs of ADPD related genes. Two non-sense mutations, c.297C > A (p.Y99X) of *CHCHD2* and c.4C > T (p.Q2X) of *TMEM230*, resulted in premature termination codons, which may produce truncated proteins and caused the disease through haploinsufficiency effect or non-sense mediated mRNA decay (NMD). *TMEM230* is a recently discovered PD-associated gene and encodes membrane transporter 230. The physiological function of this protein is unclear; but it can be found in vesicle structures in dopaminergic neurons of the substantia nigra and Purkinje neurons of the cerebellum; and may participate in the transport and circulation of synaptic vesicles, autophagy, neurotoxicity, and Golgi secretion. Several PD-linked *TMEM230* mutations have been reported in North American (Deng et al., 2016), Caucasian (Giri et al., 2017), and Chinese populations (Fan et al., 2017; Yang et al., 2017). However, functional studies are still required to determine the role of these variants. The CHCHD2 protein is located in the mitochondrial intermembrane space and is highly expressed in dopaminergic neurons of the substantia nigra. This protein is involved in the mitochondrial-mediated apoptotic pathway and regulates the function of complex IV in the mitochondrial respiratory chain (Imai et al., 2019). Abl2 kinase phosphorylation of CHCHD2 at Tyr99 is involved in the regulation of complex IV (Aras et al., 2017); the c.297C > A (p.Y99X) mutation identified in our study may be pathogenic by affecting CHCHD2 phosphorylation. There is a high linkage disequilibrium between the two mutations of *DNAJC13*, c.2578C > T (p.R860C) and c.2564A > G (p.N855S), which were reported to be the pathogenic variants of a large four-generation ADPD family (Vilariño-Güell et al., 2014). There may be synergism between these two mutations, affecting the endosomal transport and sorting function of the DNAJC13 protein, and participating in the deposition of α-synuclein aggregates and Lewy bodies in specific brain regions (Vilariño-Güell et al., 2014). Point mutations in *SNCA* may upregulate α-synuclein expression (Cronin et al., 2009), alter its solubility (Porcari et al., 2015), promote early oligomerization and accelerate α-synuclein fibrillation (Mohite et al., 2018). The c.14T > C (p.M5T) mutation in *SNCA* detected in our study may result in two possible pathogenic mechanisms. The first may be a gain-of-function (GoF) effect, resulting in increased oligomerization of α-synuclein, while the second may be a dominant negative (DN) effect, with compensatory over-expression of wild-type single-stranded DNA of the heterozygous variant. However, functional studies will be required to validate the pathogenic role of *SNCA* c.14T > C (p.M5T). The clinical manifestation of this patient is consistent with the phenotypic characteristics of missense mutation in *SNCA*: the age of onset is late (50 years old), the progress is rapid, with the development of obvious cognitive impairment (MMSE 24 points, MoCA 17 points) and mental symptoms (hallucinations, spousal infidelity delusions) after 2 years.

Finally, we report here the first case of *LRRK2* c.6055G > A (p.G2019S) mutation in a Chinese population. This is a 48-year-old man with no family history of PD. His symptoms are characterized by rigidity and bradykinesia of both lower limbs with no obvious tremor; increased axial muscular tension; Pisa syndrome; few non-motor symptoms; slow disease progression and good response to piribedil. These are consistent with the clinical phenotype of Ashkenazi Jews with *LRRK2* c.6055G > A (p.G2019S) mutations where carriers had longer disease duration; lower extremity onset; postural instability and gait difficulty; persistent response to levodopa for more than 5 years (Alcalay et al., 2013). The mutation frequency of this locus is high in North African Arabs and German Jews, but the positive rate in Eurasian populations is only 0.1%–4% (Lesage et al., 2006; Ozelius et al., 2006). Our detection of the first Chinese case with this mutation appears to be in line with these reported statistics; reinforcing the need for a deeper understanding of these population differences and their implications for diagnosis and treatment strategies.

There are several limitations in this study: first, the first identified case of *LRRK2* c.6055G > A (p.G2019S) mutation in the Chinese population can benefit from more in-depth genetic analysis. Second, the four novel P/LP variants require functional experiments to clarify their pathogenic mechanism. Finally, we only detected rare mutations in the coding region and splicing site of known pathogenic genes; evaluation of non-coding regions may reveal more pathogenic variants. With whole genome sequencing combined with MLPA technology, this may become less challenging, and comprehensive mapping of the mutation spectrum of PD will be possible.

## Conclusion

The mutation spectrum of EOPD patients in eastern China displayed different characteristics compared to previously reported populations. Larger scale epidemiological investigations across centers and regions, as well as in-depth mechanistic studies, will further improve our understanding of the etiology of EOPD. The EOPD subtyping system with a combination of clinical markers such as clinical phenotype, imaging and abnormal protein expression will facilitate early diagnosis, guide individualized treatment, and be an important means to realize clinical translation of genetic research.

## Data Availability Statement

According to national legislation/guidelines, specifically the Administrative Regulations of the People’s Republic of China on Human Genetic Resources (http://www.gov.cn/zhengce/content/2019-06/10/content_5398829.htm, http://english.www.gov.cn/policies/latest_releases/2019/06/10/content_281476708945462.htm), no additional raw data are available at this time. Data of this project can be accessed after an approved application to the China National Genebank (CNGB, https://db.cngb.org/cnsa/). Please refer to https://db.cngb.org/, or email: CNGBdb@cngb.org for detailed application guidance. The accession code CNP0002762 should be included in the application.

## Ethics Statement

The studies involving human participants were reviewed and approved by the Ethics Committee of Nanjing Medical University. The patients/participants provided their written informed consent to participate in this study.

## Author Contributions

PH and JG: conceptualization. YZ, QZ, and PH: data curation and formal analysis. PH, LL, and JR: investigation. PH: writing – original draft. JG, BT, and WL: supervision and writing – review and editing. WL: funding acquisition. BT and WL: resources. All authors contributed to the article and approved the submitted version.

## Conflict of Interest

The authors declare that the research was conducted in the absence of any commercial or financial relationships that could be construed as a potential conflict of interest.

## Publisher’s Note

All claims expressed in this article are solely those of the authors and do not necessarily represent those of their affiliated organizations, or those of the publisher, the editors and the reviewers. Any product that may be evaluated in this article, or claim that may be made by its manufacturer, is not guaranteed or endorsed by the publisher.

## Footnotes

1
http://cadd.gs.washington.edu

2
http://genetics.bwh.harvard.edu/pph2

3
http://sift.jcvi.org

4
http://www.mutationtaster.org

5
https://www.ncbi.nlm.nih.gov/pubmed/

6
http://www.genemed.tech/gene4pd/

7
https://www.mdsgene.org/g4d
