# Supplementary material for: Genetic Analysis of Patients With Early-Onset Parkinson’s Disease in Eastern China
Source: Front Aging Neurosci. 2022 May 11;14:849462. doi: 10.3389/fnagi.2022.849462 (PMC9131032; doi:10.3389/fnagi.2022.849462)
Supplement: Supplementary file 1 [file Data_Sheet_1.PDF]

Supplementary Table 1
24targeted genes in this study

Gene symbol	Gene name	Mode of Inheritance	Location	PARKs
SNCA	α-synuclein	AD	4q22.1	PARK1/4
PRKN	Parkin RBR E3 Ubiquitin Protein Ligase	AR	6q26	PARK2
UCHL1	Ubiquitin c-terminal hydrolase	AD	4p13	PARK5
PINK1	PTEN-induced putative kinase 1	AR	1p36	PARK6
DJ-1	Oncogene DJ-1	AR	1p36.23	PARK7
LRRK2	Leucine rich repeat kinase 2	AD	12q12	PARK8
ATP13A2	ATPase, type 13A2	AR	1p36.13	PARK9
GIGYF2	GRB10- interacting GYF protein 2	AD	2q37.1	PARK11
HTRA2	HTRA serine peptidase 2	AD	2p13.1	PARK13
PLA2G6	Phospholipase A2	AR	22q13.1	PARK14
FBXO7	F-box only protein 7	AR	22q12.3	PARK15
VPS35	Vacuolar protein sorting 35	AD	16q11.2	PARK17
EIF4G1	Eukaryotic translation initiation factor 4 gamma 1	AD	3q27.1	PARK18
DNAJC6	DNAJ/HSP40 homolog subfamily C member 6	AR	1p31.3	PARK19
SYNJ1	Synaptojanin 1	AR	21q22.1	PARK20
DNAJC13	DNAJ/HSP40 homolog subfamily C member 13	AD	3q22.1	PARK21
TMEM230	Transmembrane protein 230	AD	20p13	PARK21
CHCHD2	Coiled-coil-helix-coiled-coil-helix domain-containing protein	AD	7p11.2	PARK22
VPS13C	Vacuolar protein sorting 13C	AR	15a22.2	PARK23
POLG	DNA Polymerase Gamma, Catalytic Subunit	AD	15q26.1	-
LRP10	Low density lipoprotein receptor-related protein 10	AD	11p11.2	-
RIC3	RIC3 acetylcholine receptor chaperone	AD	11p15.4	-
RAB39B	Ras-associated protein RAB39B DNA Polymerase Gamma	XR	Xq28	-
GBA	Glucocerebrocidase Beta	AD	1q22	-

Supplementary Table 2

Lists of patients carrying unidentified variants in ADPD-associated genes

Sample ID	Gender	OA	Gene	Nucleotide change	A.A. alteration	Exonic Func.	Hom / Het	Mode of Inheritance	ACMG	Reported
4325	M	35	*GIGYF2*	c.2053G>A	p.V685M	nonsyn	Het	AD	US	No
4309	M	23	*DNAJC13*	c.6212G>A	p.R2071Q	nonsyn	Het	AD	US	No
4320	M	44	*LRRK2*	c.3974G>A	p.R1325Q	nonsyn	Het	AD	US	Yes
4341	F	37	*LRRK2*	c.5000T>G	p.L1667W	nonsyn	Het	AD	US	No

OA, onset age;

Supplementary Table 3

Population frequency and pathogenicity prediction of unidentified variants in ADPD-associated genes

	GIGYF2	*DNAJC13*	*LRRK2*	*LR*[illegible]
Nucleotide change	c.2053G>A	c.6212G>A	c.3974G>A	c.5000T[illegible]
A.A. alteration	p.V685M	p.R2071Q	p.R1325Q	p.L166[illegible]
Exonic Func.	nonsyn	nonsyn	nonsyn	no[illegible]
Hom/Het	Het	Het	Het	[illegible]
Mode of Inheritance	AD	AD	AD	[illegible]
gnomAD_exome_EAS	0.0002	0.0003	5.81E-05	[illegible]
ExAC_EAS	0.0001	0.0006	0.0001	[illegible]
CADD	33:D	22.7:D	34:T	27[illegible]
ReVe	0.725:D	0.788:D	0.902:D	0.92[illegible]
SIFT	0.01:D	0.627:T	0.094:T	0.00[illegible]
PolyPhen2-HVAR	0.926:D	0.023:B	0.977:D	0.99[illegible]
Mutation taster	disease causing	disease causing	disease causing	disease cau[illegible]
PhyloP	2.526:Conserved	-2.823:Nonconserved	-0.393:Nonconserved	-0.559:Nonconse[illegible]
PhastCons	1:Conserved	0.002:Nonconserved	0.836:Nonconserved	0.692:Nonconse[illegible]

Supplementary Table 4

Lists of 18 patients carrying single heterozygous variants in ARPD-associated genes

Sample ID	Gender	OA	Gene	Nucleotide change	A.A. alteration	Exonic Func.	Hom/ Het	Mode of Inheritance	Reported
4340	F	50	*PINK1*	c.1342G>A	p.G448R	nonsyn	Het	AR	No
4330	F	47	*ATP13A2*	c.2966G>C	p.R989P	nonsyn	Het	AR	No
4434	M	49	*DNAJC6*	c.674G>A	p.R225Q	nonsyn	Het	AR	No
4432	M	38	*PRKN*	c.1285+2T>C		splicing	Het	AR	No
4334	F	39	*PRKN*	c.850G>C	p.G284R	nonsyn	Het	AR	Yes
4356	M	35	*PRKN*	Exon2-3 del		CNV	Het	AR	Yes
4410	M	15	*PRKN*	Exon8-9 del		CNV	Het	AR	Yes
4344	F	44	*VPS13C*	c.9886delC	p.R3296Gfs*2	frameshift del	Het	AR	No
4404	F	50	*VPS13C*	c.9326A>G	p.Y3109C	nonsyn	Het	AR	No
4353	F	38	*VPS13C*	c.9172C>T	p.R3058C	nonsyn	Het	AR	No
4396	M	47	*VPS13C*	c.7763G>T	p.C2588F	nonsyn	Het	AR	No
			VPS13C	c.7762T>A	p.C2588S	nonsyn	Het	AR	No
4343	F	46	*VPS13C*	c.2271A>C	p.Q757H	nonsyn	Het	AR	No
4380	F	46	*VPS13C*	c.1866C>A	p.D622E	nonsyn	Het	AR	No
4389	M	48	*VPS13C*	c.1867C>A	p.D623E	nonsyn	Het	AR	No
4415	M	45	*SYNJ1*	c.2402_2405del	p.N801Rfs*11	frameshift del	Het	AR	No
4387	M	32	*SYNJ1*	c.1652C>T	p.A551V	nonsyn	Het	AR	No
4392	M	50	*SYNJ2*	c.2134G>A	p.A712T	nonsyn	Het	AR	No
4302	M	43	*SYNJ1*	c.329G>A	p.G110D	nonsyn	Het	AR	No

Note: It was confirmed by BAM file that the two variants of patient No. 4396 were on the same chromosome, so they were simple heterozygous variants

Supplementary Table 5

Comparation of the clinical phenotypes between carriers with AD/AR gene variants and non-carrier

Clinical features	Non-carriers （n=122）	AD gene variants （n=6）	*P*	AR gene variants （n=8）	*P*
Age at onset (year)*	44.48±5.76	43.83±8.95	0.374	30.25±9.07	**0.000**
Male (case, %)	71（58.2%）	4（66.7%）	0.694	7（87.5%）	0.257
Age at assessment (year)*	51.61±8.18	48.38±12.05	0.915	40.81±14.01	0.069
Duration at assessment (year)*	5.0（1-50）	5.0（2-12）	0.491	11.25（2-31）	0.447
LED (mg/d)	515.0±279.8	449.0±164.6	0.236	387.0±170.3	0.413
UPDRS-I score	3.48±2.59	4.25±0.96	0.555	4.00±3.00	0.664
ADL score*	12.92±7.22	9.75±2.06	0.383	15.40±12.99	0.768
UPDRS-III score	29.30±16.17	23.20±15.60	0.425	32.00±18.05	0.841
H-Y*	2.10±0.87	1.83±0.52	0.546	2.25±1.54	0.775
MMSE score*	27.54±2.48	27.25±2.75	0.790	27.33±2.94	0.930
MoCA score*	23.49±5.07	23.25±4.92	0.837	24.83±5.46	0.372
HAMD score	11.08±7.31	9.75±4.92	0.720	9.14±6.20	0.641
HAMA score*	7.54±5.38	6.25±2.63	0.642	5.17±3.87	0.499
PDSS score	120.18±21.99	130.50±13.20	0.359	134.0±16.97	0.383
NMSQ score	9.37±5.41	10.00±4.16	0.821	6.20±1.00	**0.003**

Supplementary Methods

Some pathogenic variants were verified using sanger sequencing, and related primer sequences were as follows:

PRKN (NM_004562.3:exon7:c.850G>C:p.G284R):

Forward:5'-CAATTCCTTCATTCCCCAGA-3'

Reverse5'-TAATGCTTAGCAGCTCCGGT-3'

PRKN (NM_004562.3:exon9:c.1079G>T:p.C360F):

Forward:5'- GTGGCTCACAGCATCTTAAAGT-3'

Reverse5'- CCCCTACACACACACACACA-3'

LRRK2 (NM_198578.4:exon41:c.6055G>A:p.G2019S)

Forward:5'- AAGGGACAAAGTGAGCACAGA-3'

Reverse5'- TCAGTTTTTGCCCTGAAAAATTACA-3'

CHCHD2 (NM_016139.4:exon2:c.297C>A:p.Y99X)

Forward:5'- CTCCTCCTTGGGAAGAAACACA-3'

Reverse5'- TCAGTTTTTGCCCTGAAAAATTACA-3'

*The patient No.4355 (PLA2G6 c.1634A>G, c.991G>T) once performed genetic testing in the biological company by himself, but has now lost the visit and is unable to provide primer sequences.

Supplementary Figure1

Pedigree chart of 9 Parkinson's disease families with P/LP variants in PD associated genes

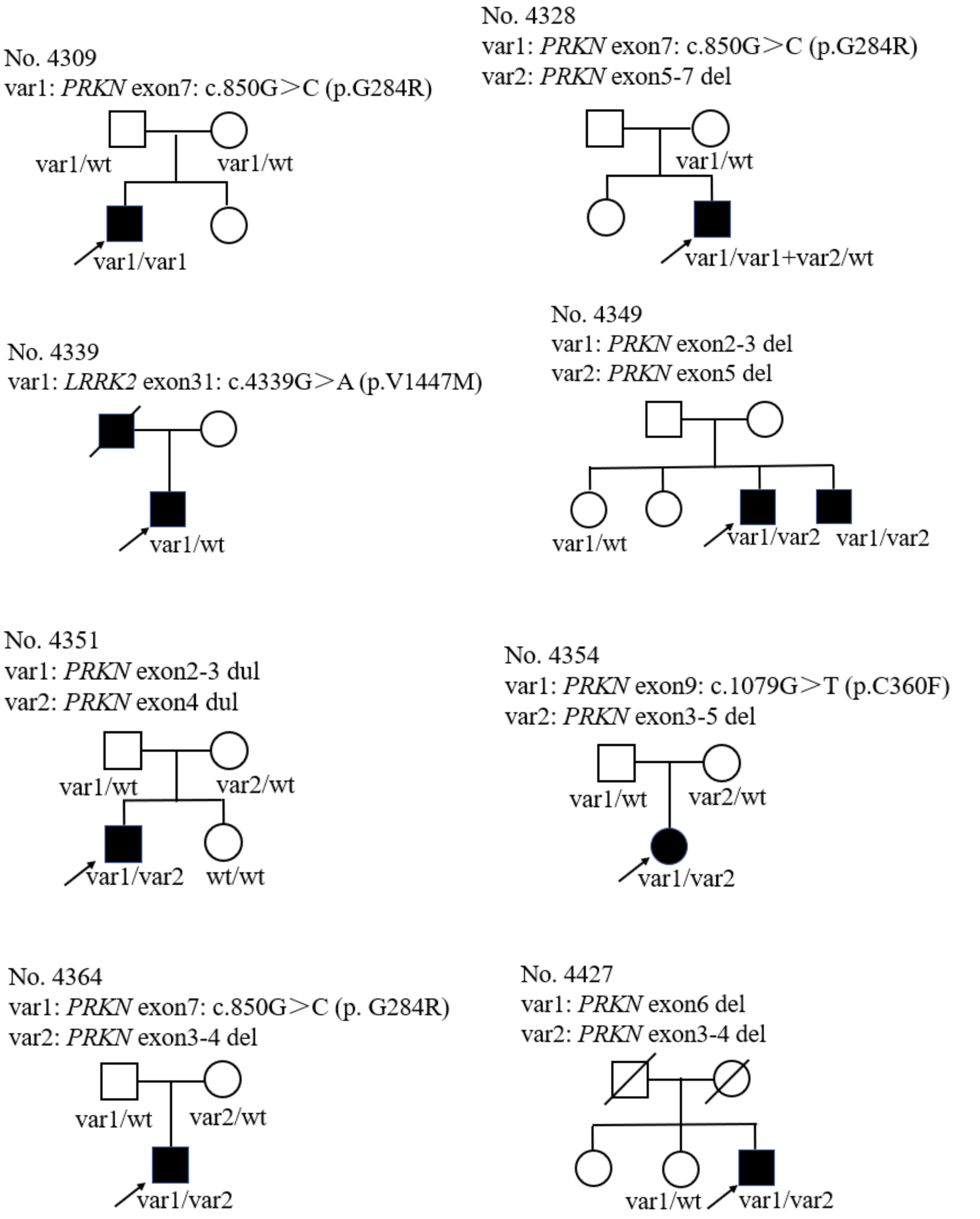

No. 4355
var1: *PLA2G6* exon12: c.1634A>G (p.K545R)
var2: *PLA2G6* exon7: c.991G>T (p.D331Y)

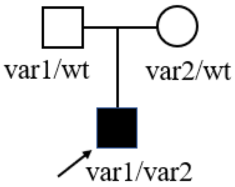

Legend: var, variant; wt, wildtype; var1/var2, means compound heterozygote; del, deletion; dup, duplication
