# Supplementary material for: Genetic Analysis of Patients With Early-Onset Parkinson’s Disease in Eastern China
Source: Front Aging Neurosci. 2022 May 11;14:849462. doi: 10.3389/fnagi.2022.849462 (PMC9131032; doi:10.3389/fnagi.2022.849462)

No. 4328 *PRKN* Exon5-7 del/ c.850G>C(hom)

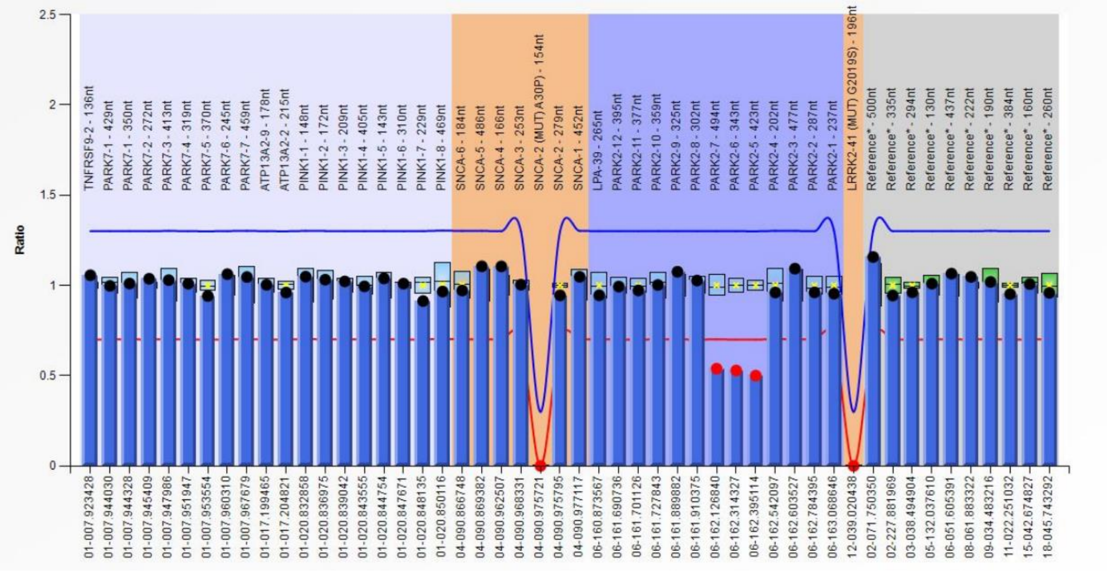

No.4349: *PRKN* Exon2-3 del/Exon5 del

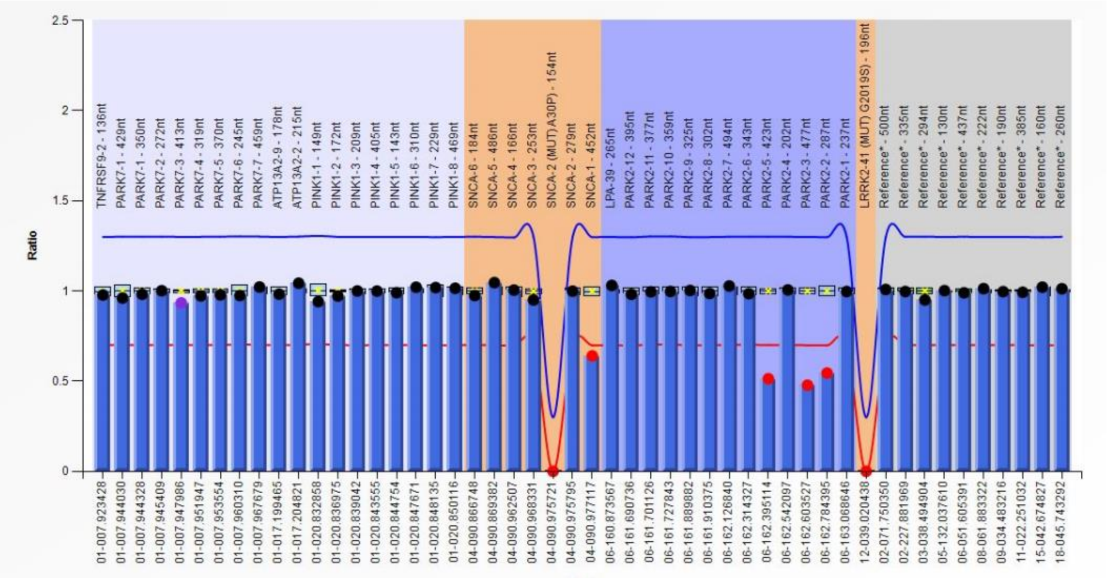

We detected the *SNCA* Exon1 of this sample by qPCR.

Sister of No.4349: *PRKN* Exon2-3 del

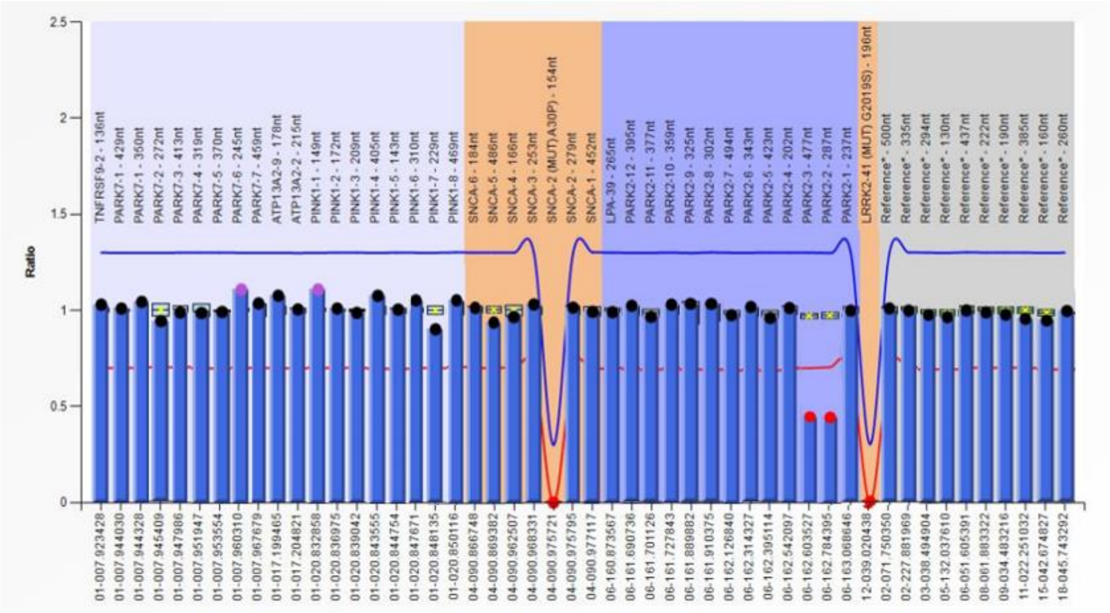

Brother of No.4349: Exon2-3 del/Exon5 del

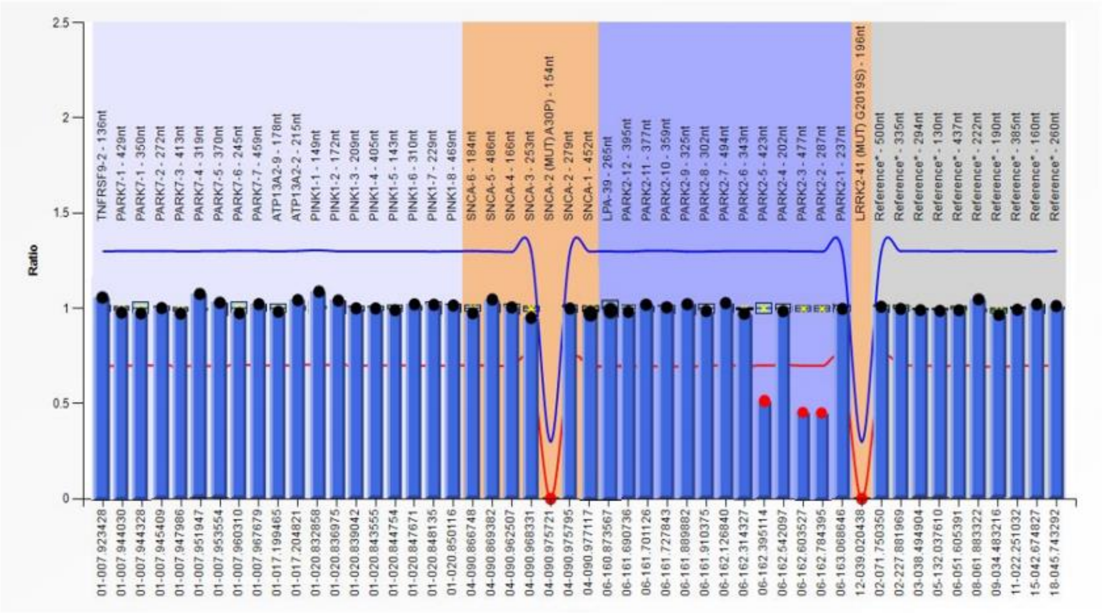

No.4351: *PRKN* Exon2-3 dul / Exon4 dul

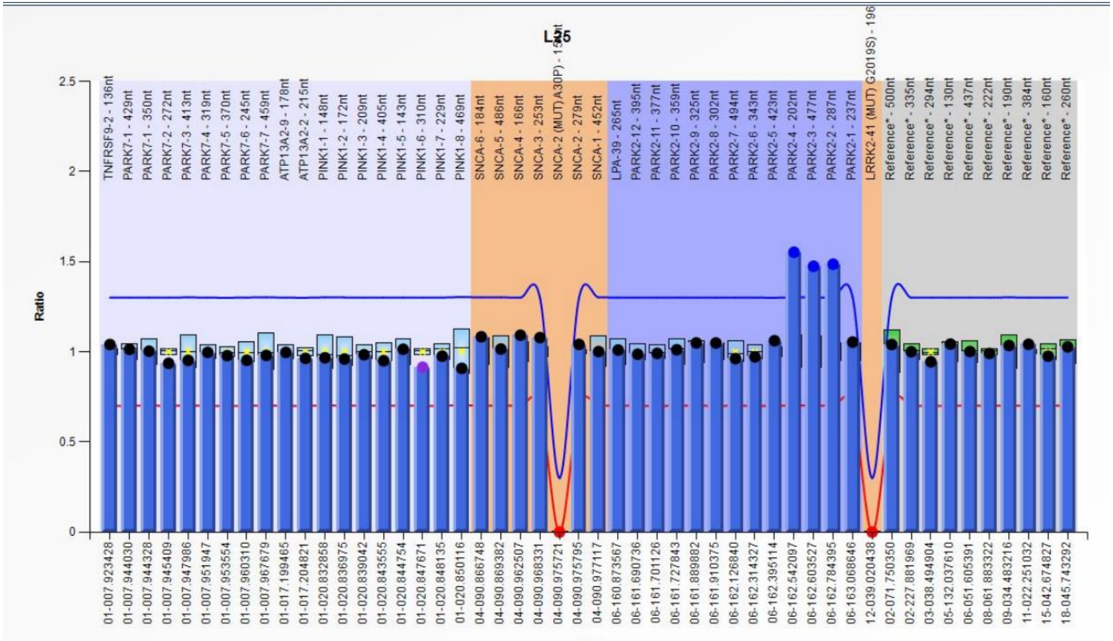

Father of No.4351 *PRKN* Exon2-3 dul

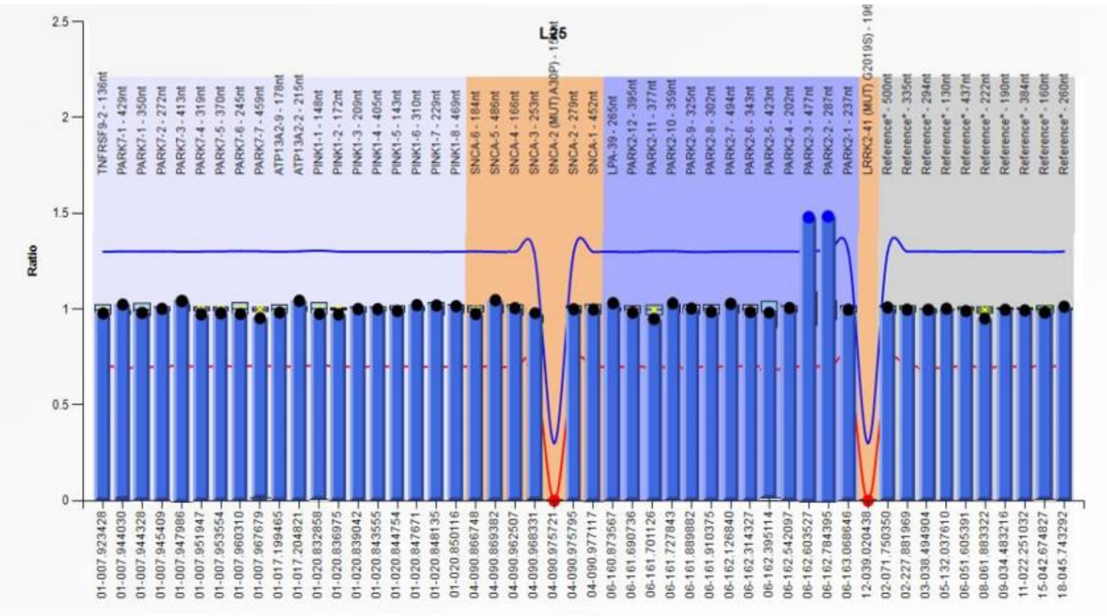

Mather of No.4351 *PRKN* Exon4 dul

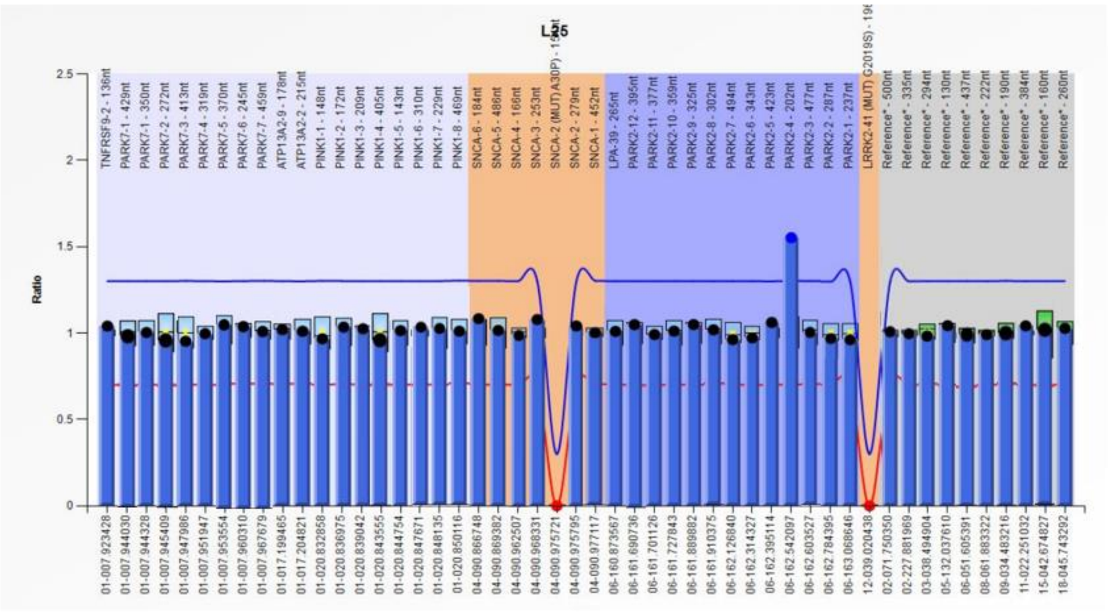

No.4354 *PRKN* Exon3-5 del/ c.1079G>T(het)

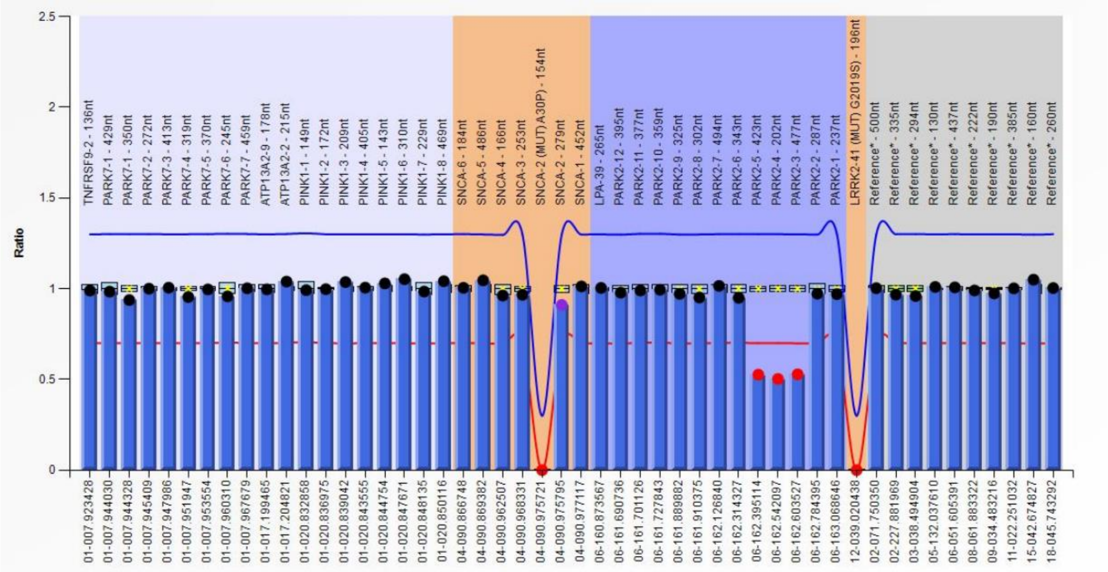

Mother of No.4354 *PRKN* Exon3-5 del

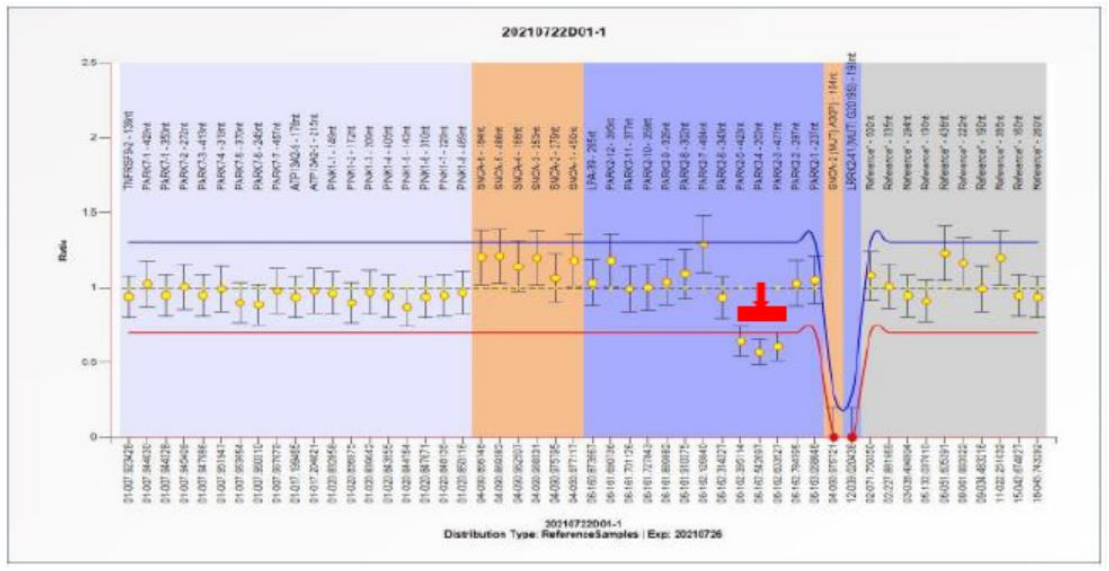

No.4356 *PRKN* Exon2-3 del

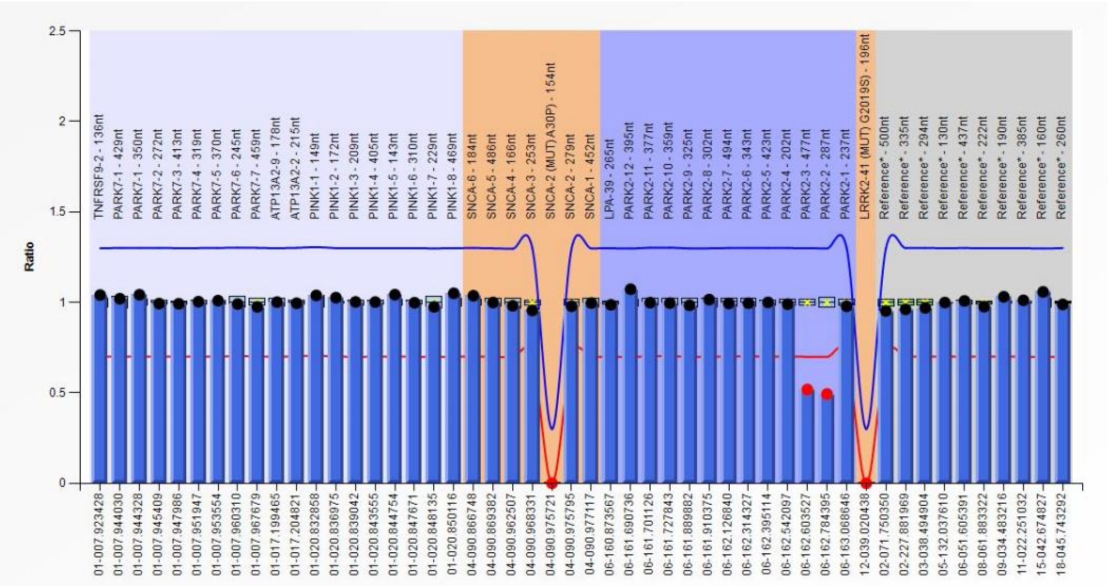

No.4364 *PRKN* Exon3-4 del / c.850G>C(het)

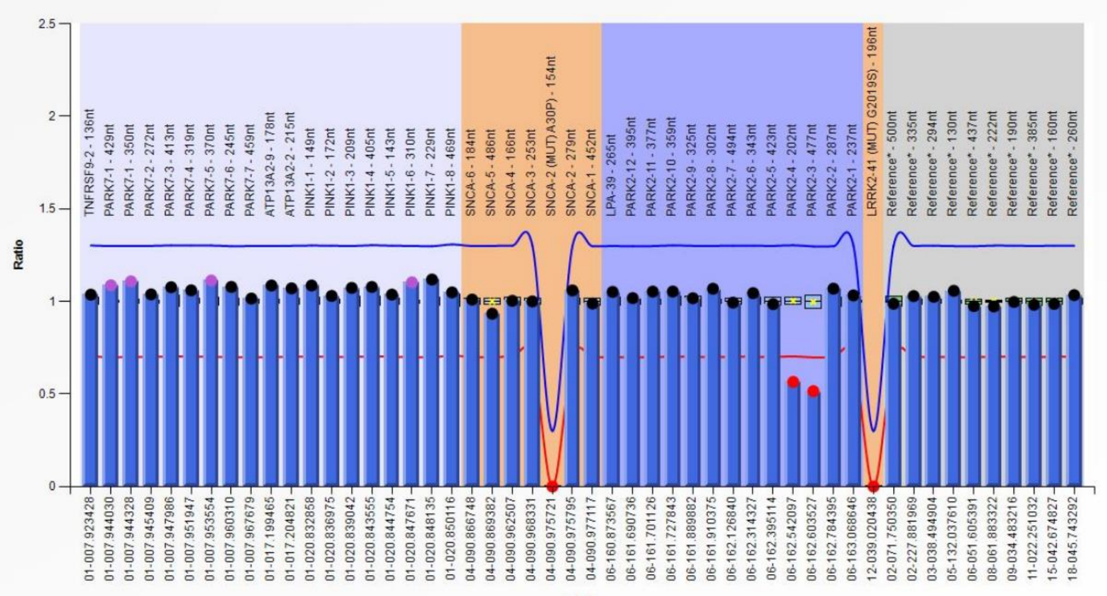

Mother of No.4364 *PRKN* Exon3-4 del

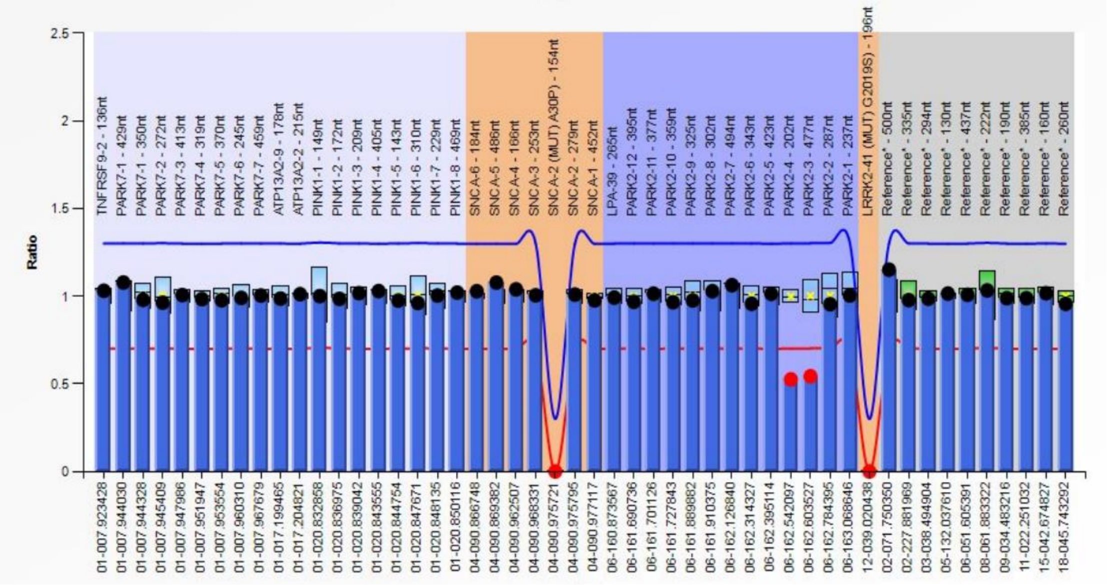

No.4427 *PRKN* Exon3-4 del/Exon6 del

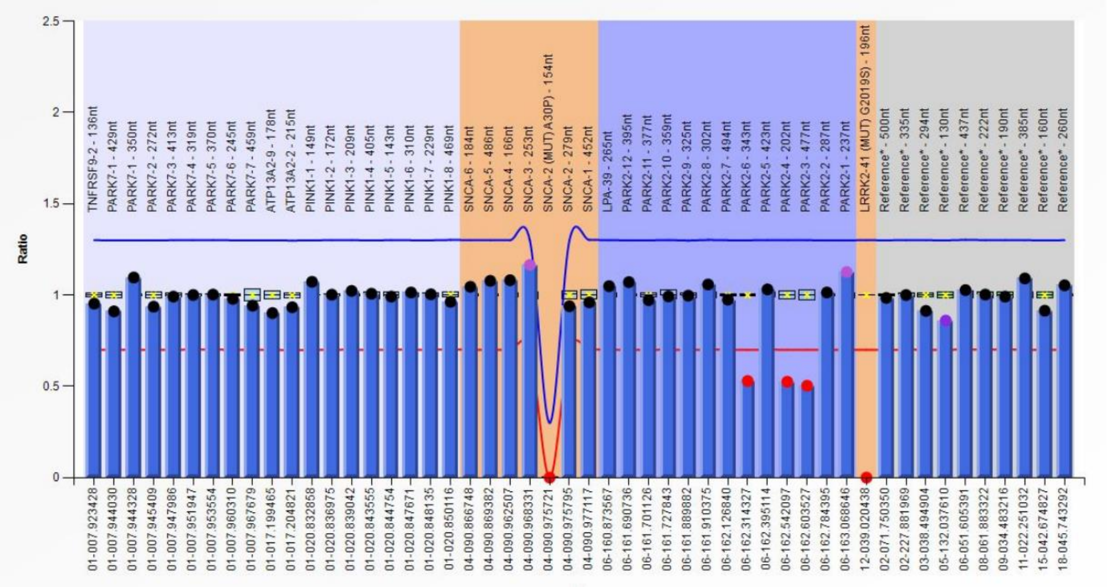

Sister of No.4427: *PRKN* Exon6 del

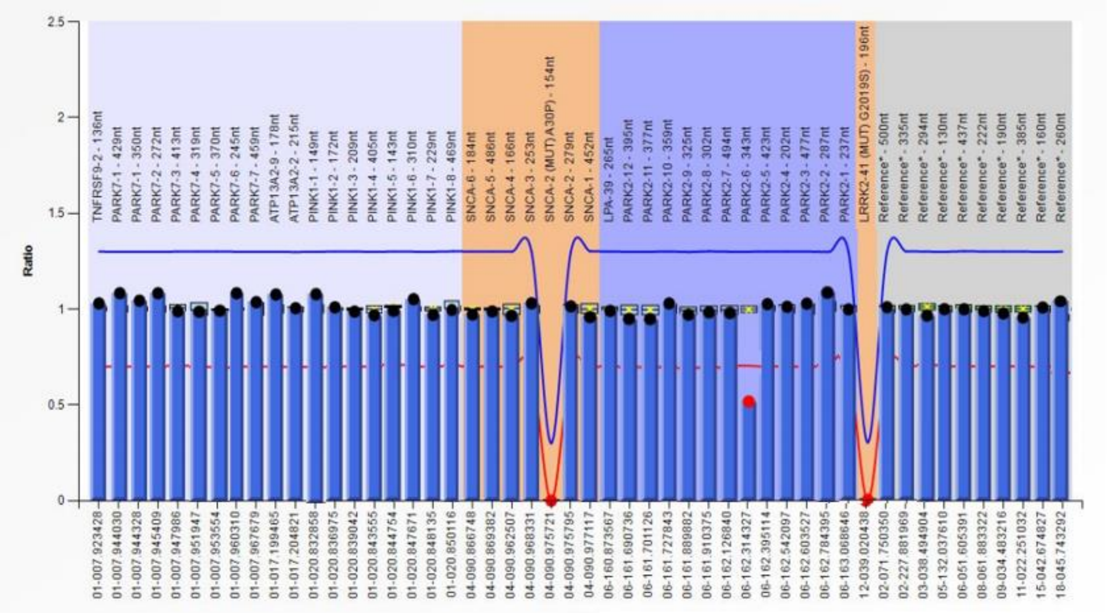

No.4410 *PRKN* Exon8-9 del

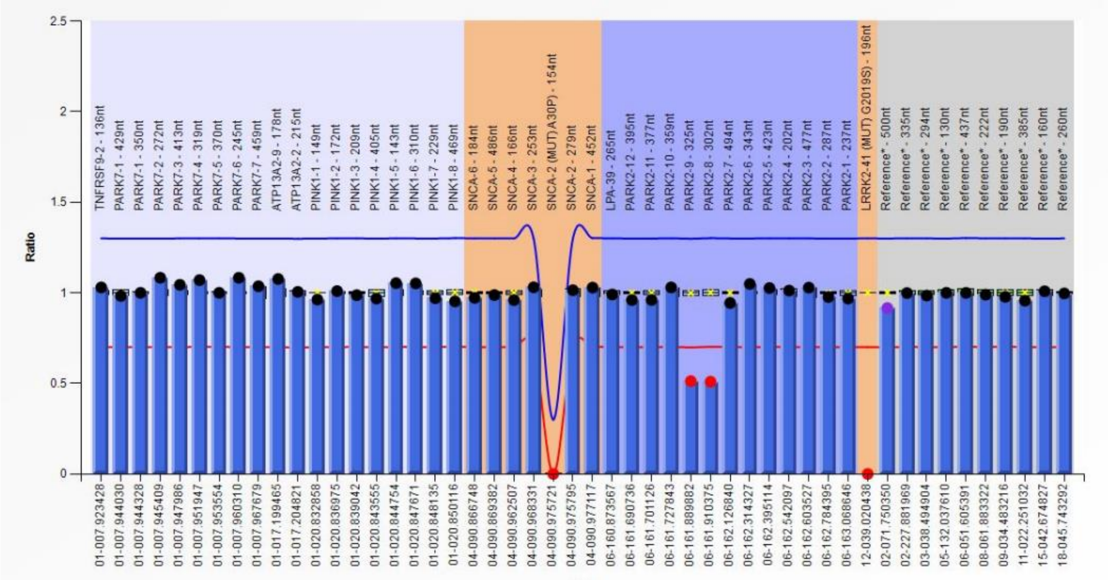

Father of No.4410 *PRKN* Exon8-9 del

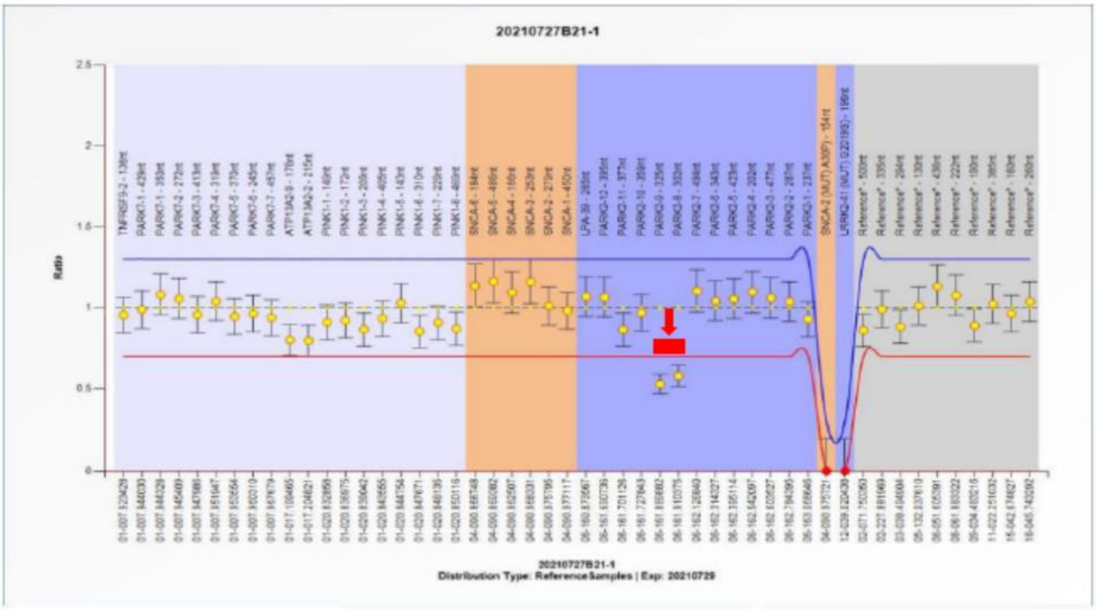

Supplement: Supplementary file 2 [file Data_Sheet_2.PDF]
